# Plasma phosphorylated tau biomarkers map onto [^18F]FDG PET hypometabolism: a voxel-wise study in a clinical cohort with CSF-confirmed AD subgroup analysis

**DOI:** 10.1007/s00259-026-07939-7

**Published:** 2026-05-27

**Authors:** Agostino Chiaravalloti, Caterina Motta, Chiara Giuseppina Bonomi, Martina Poli, Carmela Di Russo, Orazio Schillaci, Alessandro Martorana

**Affiliations:** 1 Department of Biomedicine and Prevention, University of Rome Tor Vergata, Rome, Italy; 2 IRCCS Neuromed, Pozzilli, Italy; 3 Memory Clinic and Neurodegenerative Dementia Research Unit, Policlinico Tor Vergata, University of Rome Tor Vergata, Rome, Italy; 4 Nuclear Medicine Unit, Policlinico Tor Vergata, Rome, Italy

**Keywords:** Alzheimer’s disease, [^18F]FDG PET, Statistical Parametric Mapping, Plasma biomarkers, Phosphorylated tau, p-tau181, p-tau181/Aβ42, p-tau217, Cerebellar normalisation, Voxel-wise analysis

## Abstract

**Purpose:**

To examine voxel-wise associations between plasma phosphorylated tau biomarkers and cerebellum-normalised [^18F]fluorodeoxyglucose ([^18F]FDG) positron emission tomography (PET) uptake in cognitively impaired patients, and whether these associations persist in a cerebrospinal fluid (CSF)-confirmed Alzheimer’s disease (AD) subgroup.

**Methods:**

One hundred consecutive patients who underwent brain [^18F]FDG PET and plasma biomarker assessment within ≤ 6 months were included. Images were spatially normalised, smoothed, and divided voxel-wise by a subject-specific bilateral cerebellar reference value derived from Automated Anatomical Labelling 3 (AAL3) cerebellar regions. Voxel-wise multiple regressions tested associations with plasma Aβ42/Aβ40, p-tau181, p-tau181/Aβ42, p-tau217, and p-tau217/Aβ42, adjusting for age and sex, in the full cohort and the CSF-confirmed AD subgroup (*n* = 58).

**Results:**

In the full cohort, plasma p-tau181 and p-tau181/Aβ42 showed negative associations with cerebellum-normalised [^18F]FDG uptake, predominantly in bilateral ventral temporal/fusiform regions. The strongest and most spatially consistent finding involved p-tau181/Aβ42. Plasma Aβ42/Aβ40 and p-tau217 showed no suprathreshold associations, whereas p-tau217/Aβ42 showed only a small focal cluster. In the CSF-confirmed AD subgroup, p-tau181/Aβ42 remained the most consistent tau-related marker, showing negative associations with cerebellum-normalised [^18F]FDG uptake in temporal/temporo-limbic and posterior cortical regions, whereas p-tau217 and p-tau217/Aβ42 showed no suprathreshold clusters.

**Conclusions:**

After voxel-wise cerebellar normalisation, plasma p-tau181/Aβ42 showed the most robust association with [^18F]FDG PET hypometabolism, predominantly involving bilateral ventral temporal/fusiform regions in the full cohort and temporal/temporo-limbic and posterior cortical regions in the CSF-confirmed AD subgroup. The heterogeneous full-cohort findings may partly reflect AD-versus-non-AD biological separation. Overall, plasma tau biomarkers and [^18F]FDG PET provide complementary molecular and metabolic information.

**Supplementary information:**

The online version contains supplementary material available at 10.1007/s00259-026-07939-7.

## Introduction

Alzheimer’s disease (AD) diagnostics are increasingly shifting from a purely clinical, syndrome-based approach to a biologically anchored framework in which amyloid-β and tau pathology are documented with biomarkers and complemented by measures of neurodegeneration and synaptic dysfunction. In this context, blood-based biomarkers (BBMs) represent a major opportunity: compared with cerebrospinal fluid (CSF) biomarkers and positron emission tomography (PET) imaging, plasma assays are minimally invasive, potentially less expensive, and inherently scalable, with clear utility for routine work-up, prescreening, and trial enrichment [1]. Among BBMs, plasma phosphorylated tau species—particularly p-tau181 and p-tau217—have demonstrated clinically meaningful performance for identifying AD pathophysiology and for risk stratification. In preclinical AD, plasma p-tau217 predicted longitudinal cognitive decline and improved the explained variance of cognitive slopes beyond covariates alone, with validation in an independent cohort [2]. [^18F]fluorodeoxyglucose ([^18F]FDG) PET provides a well-established readout of regional cerebral glucose metabolism, reflecting neuronal/synaptic dysfunction and yielding characteristic spatial patterns in AD [3]. [^18F]FDG PET is therefore clinically useful both for differential diagnosis and for capturing the functional downstream expression of AD pathology [4]. Conceptually, plasma biomarkers and [^18F]FDG PET should be related, as circulating amyloid- and tau-linked measures index core molecular pathology, whereas [^18F]FDG PET captures functional impairment that may track with disease stage and neurodegenerative burden [5].

However, despite the rapid expansion of the BBM field, studies that directly integrate plasma AD biomarkers with [^18F]FDG PET remain limited and heterogeneous, which weakens interpretability at the individual-patient level. A recent Alzheimer’s Disease Neuroimaging Initiative (ADNI)-based comparison explicitly quantified the diagnostic performance of plasma p-tau181 versus [^18F]FDG PET for identifying biologically defined AD. When classifying CSF-positive versus CSF-negative AD biomarker status across the overall sample, plasma p-tau181 achieved an area under the curve (AUC) of 0.80, whereas [^18F]FDG PET standardized uptake value ratio (SUVR) achieved an AUC of 0.73 [6]. Importantly, the separation was most evident in mild cognitive impairment (MCI), where plasma p-tau181 reached an AUC of 0.78 compared with [^18F]FDG PET AUC 0.66 for the same CSF-defined outcome [6]. Using amyloid PET as reference in probable AD, plasma p-tau181 also showed higher discrimination than [^18F]FDG PET [6]. At the same time, [^18F]FDG PET uptake related more strongly to cognitive outcomes than plasma p-tau181, underscoring that these modalities are not interchangeable and likely capture different portions of the disease cascade [6]. Complementary evidence comes from a tertiary-care cohort enriched for early-onset and/or atypical dementia, where plasma p-tau217 was directly compared with expert visual reads of [^18F]FDG PET. For identifying an AD CSF biomarker profile, plasma p-tau217 showed higher overall accuracy than [^18F]FDG PET, with markedly higher sensitivity at similar specificity [7]. These results support the view that plasma p-tau—especially p-tau217—may detect biological AD even when hypometabolism patterns are not yet clearly expressed or are confounded by atypical presentations; conversely, [^18F]FDG PET remains tightly linked to the clinical phenotype [7]. Beyond head-to-head accuracy, multimodal work in symptomatic AD has started to map how plasma markers relate to imaging measures. In a mild-to-moderate AD cohort, plasma p-tau181 and glial fibrillary acidic protein (GFAP) correlated with tau PET, whereas neurofilament light chain (NfL) correlated with [^18F]FDG PET in regions such as the precuneus, indicating that different plasma biomarkers may capture different biological dimensions of AD [8].

Despite these encouraging results, current evidence remains dominated by classification-oriented studies reporting global diagnostic metrics, whereas fewer data are available on the spatial relationship between plasma tau biomarkers and regional metabolic dysfunction on [^18F]FDG PET. In particular, it remains unclear whether plasma tau biomarkers converge on a common AD-vulnerable metabolic network across clinically heterogeneous populations, and whether these associations are maintained after restricting analyses to biologically confirmed AD.

In our previous work, we investigated associations between [^18F]FDG PET metabolism and AD biomarkers measured in CSF [9]. Building on this rationale, the present study focused on plasma tau biomarkers and examined the voxel-wise relationships between regional [^18F]FDG PET metabolism and plasma p-tau181, p-tau181/Aβ42, p-tau217, and p-tau217/Aβ42 in a real-world clinical cohort of patients with cognitive impairment. Given the known limitations of whole-brain proportional scaling in dementia [^18F]FDG PET studies, voxel-wise analyses were performed on cerebellum-normalised [^18F]FDG images. We further tested whether the observed associations persisted in a CSF-confirmed AD subgroup. We hypothesised that plasma tau-related biomarkers would show negative associations with cerebellum-normalised [^18F]FDG uptake in AD-relevant cortical regions, and that the strength and topography of these associations would differ between the clinically heterogeneous full cohort and the biologically confirmed AD subgroup.

## Materials and methods

Between January 2022 and April 2025, we enrolled 175 consecutive patients evaluated at the UOSD Centro Demenze, University Hospital “Policlinico Tor Vergata” in Rome. Patients presenting with cognitive complaints underwent a comprehensive diagnostic work-up aimed at investigating an underlying neurodegenerative process, including neuropsychological assessment, laboratory testing, brain magnetic resonance imaging (MRI), and [^18F]FDG PET imaging. Structural MRI findings were reviewed as part of the routine multidisciplinary diagnostic work-up, and patients with gross structural abnormalities potentially interfering with the interpretation of metabolic imaging were excluded.

Cognitive status was assessed as part of the routine clinical work-up, including Mini-Mental State Examination (MMSE). Structural MRI findings were reviewed within the multidisciplinary diagnostic evaluation.

Exclusion criteria for the present study were: 1) refusal to undergo lumbar puncture or presence of anatomical/pharmacological contraindications (e.g., anticoagulant therapy, previous lumbar stabilization surgery); 2) > 6 months distance between lumbar puncture and [^18F]FDG PET; 3) alternative systemic or metabolic conditions that could account for cognitive decline; 4) decompensated diabetes mellitus type 2 altering [^18F]FDG PET imaging. No additional exclusions were applied solely on the basis of MRI findings beyond the routine clinical and diagnostic exclusion process.

### CSF, blood sampling and biomarkers analysis

All lumbar punctures were performed between 8 and 10 am. Approximately 8 mL of CSF were collected into polypropylene tubes and processed according to the laboratory’s standard operating procedures. Of the total volume, 2 mL were used for routine biochemical analysis, including cell and protein count, whereas the remaining 6 mL were centrifuged at 2000 g at + 4 °C for 10 min, aliquoted into 1 mL portions, and stored at -80 °C until further analysis. Blood samples were obtained within ± 30 min from lumbar punctures and centrifuged at 3500 g at + 4 °C for 10 min. Plasma was then aliquoted and frozen with the same procedures used for CSF samples. Blood samples were subjected to routine laboratory testing. CSF levels of Aβ40, Aβ42, p-tau181 and total tau, as well as plasma levels of Aβ40, Aβ42, p-tau181 and p-tau217, were measured using fully automated chemiluminescent enzyme immunoassay (CLEIA) Fujirebio LUMIPULSE^®^ G1200 (Fujirebio, Inc., Tokyo, Japan) as previously described [10].

In the present study, biologically confirmed AD was defined using the CSF p-tau181/Aβ42 ratio (≥ 0.069), in accordance with the diagnostic workflow adopted in our centre and based on prior internal analytical and clinical validation of the assay in the local laboratory setting. This operational threshold was obtained in a larger internal biomarker cohort, in which the p-tau181/Aβ42 ratio was optimized against the Aβ42/Aβ40 ratio-based amyloid classification, and was further considered in light of published evidence supporting ratio-based CSF biomarker classification [10]. This ratio-based approach was selected as a pragmatic biomarker criterion to identify cases with biologically supported AD pathology in the context of routine clinical practice. The threshold was applied retrospectively to the existing clinical cohort according to the local laboratory workflow and was not derived or optimized from the present imaging analyses. In addition, plasma p-tau181/Aβ42 and plasma p-tau217/Aβ42 ratios were calculated.

### Statistical analysis of the study population

After classifying the study population according to the presence (AD) or absence (non-AD) of AD pathological changes, we compared continuous variables using the Mann–Whitney U test and categorical variables using the chi-square test. A two-tailed p value < 0.05 was considered statistically significant. Statistical analyses were performed using jamovi (The jamovi project, 2024) [11]. Demographic and clinical variables included age, sex, MMSE corrected score, and education years.

### PET acquisition and image processing

All PET examinations were performed at the Nuclear Medicine Unit of the University Hospital “Policlinico Tor Vergata” (Rome, Italy) using a Discovery MI PET/computed tomography (PET/CT) system (GE HealthCare). Participants fasted for at least 5 h prior to tracer injection; blood glucose was measured before administration and confirmed to be within acceptable limits, consistent with standard brain [^18F]FDG PET recommendations.

Patients received an intravenous injection of [^18F]FDG (dose range 185–200 MBq), followed by hydration with 500 mL of 0.9% saline. Image acquisition started 30 min post-injection and lasted 10 min. Reconstruction and acquisition settings followed routine clinical brain [^18F]FDG PET procedures, consistent with established guidelines and as previously reported by our group [9, 12].

#### Image preprocessing and cerebellar normalisation

PET images were converted from Digital Imaging and Communications in Medicine (DICOM) to Neuroimaging Informatics Technology Initiative (NIfTI)/Analyze format using MRIcron [13] and preprocessed in Statistical Parametric Mapping version 25 (SPM25; v25.01.02) [14] running on MATLAB R2025b [15]. Spatial normalisation was performed using an in-house [^18F]FDG PET template/tissue probability map, previously developed from a large dataset of brain [^18F]FDG PET scans (285 AD patients and 121 healthy controls) and embedded within the SPM processing stream [16]. To reduce intensity inhomogeneity effects and improve automated processing, bias regularisation was applied (regularisation factor 0.0001) and the bias FWHM was constrained (upper bound 60 mm) to avoid modelling intensity differences attributable to distinct tissue classes. Images were smoothed with an 8 mm full width at half maximum (FWHM) Gaussian kernel to balance sensitivity and anatomical specificity, consistent with standard [^18F]FDG PET voxel-wise analyses. An affine registration using mutual information was used for initial alignment, followed by nonlinear warping (regularisation parameters set as a 1 × 5 array [0, 0.001, 0.5, 0.05, 0.2]; sampling distance 3 mm), in line with our previously reported pipeline.

To avoid the potential bias introduced by whole-brain proportional scaling in patients with widespread cortical hypometabolism, voxel-wise analyses were performed on cerebellum-normalised [^18F]FDG images. A bilateral cerebellar reference mask was generated in Montreal Neurological Institute (MNI) space using the Automated Anatomical Labelling 3 (AAL3) atlas available as a dedicated SPM toolbox [17]. The mask included bilateral cerebellar hemispheric regions and vermis labels. For each subject, the mean cerebellar uptake was extracted from the spatially normalised and smoothed [^18F]FDG image. A subject-specific cerebellum-normalised SUVR image was then generated by dividing each voxel by the corresponding mean cerebellar uptake value. These cerebellum-normalised SUVR images were used for all voxel-wise statistical analyses.

### Voxel-wise statistical analysis

Voxel-wise multiple regression analyses were performed in SPM25 (v25.01.02) to test the association between cerebellum-normalised regional [^18F]FDG uptake and each plasma biomarker across the full study cohort. The biomarkers tested were plasma Aβ42/Aβ40, p-tau181, p-tau181/Aβ42, p-tau217, and p-tau217/Aβ42. For each model, the biomarker of interest was entered as the main regressor, with age and sex included as nuisance covariates. Covariates were mean-centred using the overall mean, and the intercept was included.

Because the input images were already normalised to a subject-specific cerebellar reference value, no whole-brain proportional scaling or additional global normalisation was applied in the SPM models. Statistical inference in the full cohort was performed at voxel-level *p* < 0.001 uncorrected, with a minimum cluster extent of k ≥ 100 voxels. The same modelling strategy was then applied to the CSF-confirmed AD subgroup (*n* = 58). Given the smaller sample size of this subgroup, statistical maps were evaluated at voxel-level *p* < 0.01 uncorrected with a minimum cluster extent of k ≥ 1000 voxels, consistent with the exploratory subgroup framework.

## Results

The study ultimately included 100 patients. Based on CSF analysis, neuropsychological evaluation, and [^18F]FDG PET examination, patients were classified as follows: mild cognitive impairment or mild dementia due to AD (*n* = 58), behavioral variant frontotemporal dementia (*n* = 14), primary progressive aphasia (*n* = 3), dementia with Lewy bodies (*n* = 13), and primary psychiatric non-neurodegenerative disorder (*n* = 12) [18, 19]. Demographic, clinical, and laboratory characteristics of the study population are summarized in Table 1.

**Table 1:** Demographic, clinical, and laboratory characteristics of the study population stratified according to CSF-based diagnosis (AD vs. non-AD). Data are presented as median (interquartile range) for continuous variables and *n* (%) for categorical variables. AD = Alzheimer’s disease; CSF = cerebrospinal fluid; MMSE = Mini-Mental State Examination; p-tau = phosphorylated tau; t-tau = total tau. Group comparisons were performed using the Mann–Whitney U test for continuous variables and the chi-square test for categorical variables

Variable	AD (*n* = 58)	non-AD (*n* = 42)	Test statistic	*p* value
Age, years	75.1 (8.03)	73.6 (10.5)	1029.5	0.189
Male sex, n (%)	21 (36.2)	31 (73.8)	χ² = 12.33	< 0.001
MMSE corrected score	23.0 (6.3)	25.7 (3.4)	618.0	< 0.001
Education, years	8.0 (8.0)	11.0 (5.0)	889.5	0.041
CSF Aβ42/Aβ40	0.049 (0.014)	0.101 (0.008)	18.0	< 0.001
CSF p-tau181 (pg/mL)	81.20 (48.40)	29.50 (16.90)	111.0	< 0.001
CSF t-tau (pg/mL)	554.00 (323.00)	236.00 (113.00)	198.0	< 0.001
CSF p-tau181/Aβ42	0.188 (0.144)	0.032 (0.009)	0.0	< 0.001
Plasma Aβ42/Aβ40	0.081 (0.011)	0.090 (0.020)	707.5	< 0.001
Plasma p-tau181 (pg/mL)	2.19 (1.30)	1.27 (1.01)	418.0	< 0.001
Plasma p-tau181/Aβ42	0.089 (0.043)	0.036 (0.022)	176.0	< 0.001
Plasma p-tau217 (pg/mL)	0.404 (0.596)	0.101 (0.134)	402.0	< 0.001
Plasma p-tau217/Aβ42	0.015 (0.021)	0.003 (0.003)	353.0	< 0.001

### Voxel-wise cerebellum-normalised analysis in the full study cohort

In the full study cohort (*n* = 100), voxel-wise multiple regression analyses were performed using cerebellum-normalised [^18F]FDG SUVR images, with age and sex included as covariates.

No suprathreshold clusters were detected for plasma Aβ42/Aβ40 at the prespecified threshold. Plasma p-tau181 showed negative associations with cerebellum-normalised [^18F]FDG uptake in bilateral ventral temporal/fusiform regions. Two suprathreshold clusters were identified, with peak voxels in the left ventral temporal/fusiform cortex (kE = 1348; peak T = 4.50; MNI coordinates − 48, −46, − 12) and right ventral temporal/fusiform cortex (kE = 433; peak T = 4.49; MNI coordinates 44, − 54, −8).

The strongest and most spatially consistent full-cohort finding was observed for plasma p-tau181/Aβ42. Higher plasma p-tau181/Aβ42 was associated with lower cerebellum-normalised [^18F]FDG uptake in bilateral ventral temporal/fusiform regions. Two suprathreshold clusters were detected, with peaks in the left ventral temporal/fusiform cortex (kE = 3340; peak T = 5.31; MNI coordinates − 50, −44, − 14) and right ventral temporal/fusiform cortex (kE = 1883; peak T = 4.93; MNI coordinates 52, − 44, −12).

**Fig. 1:**
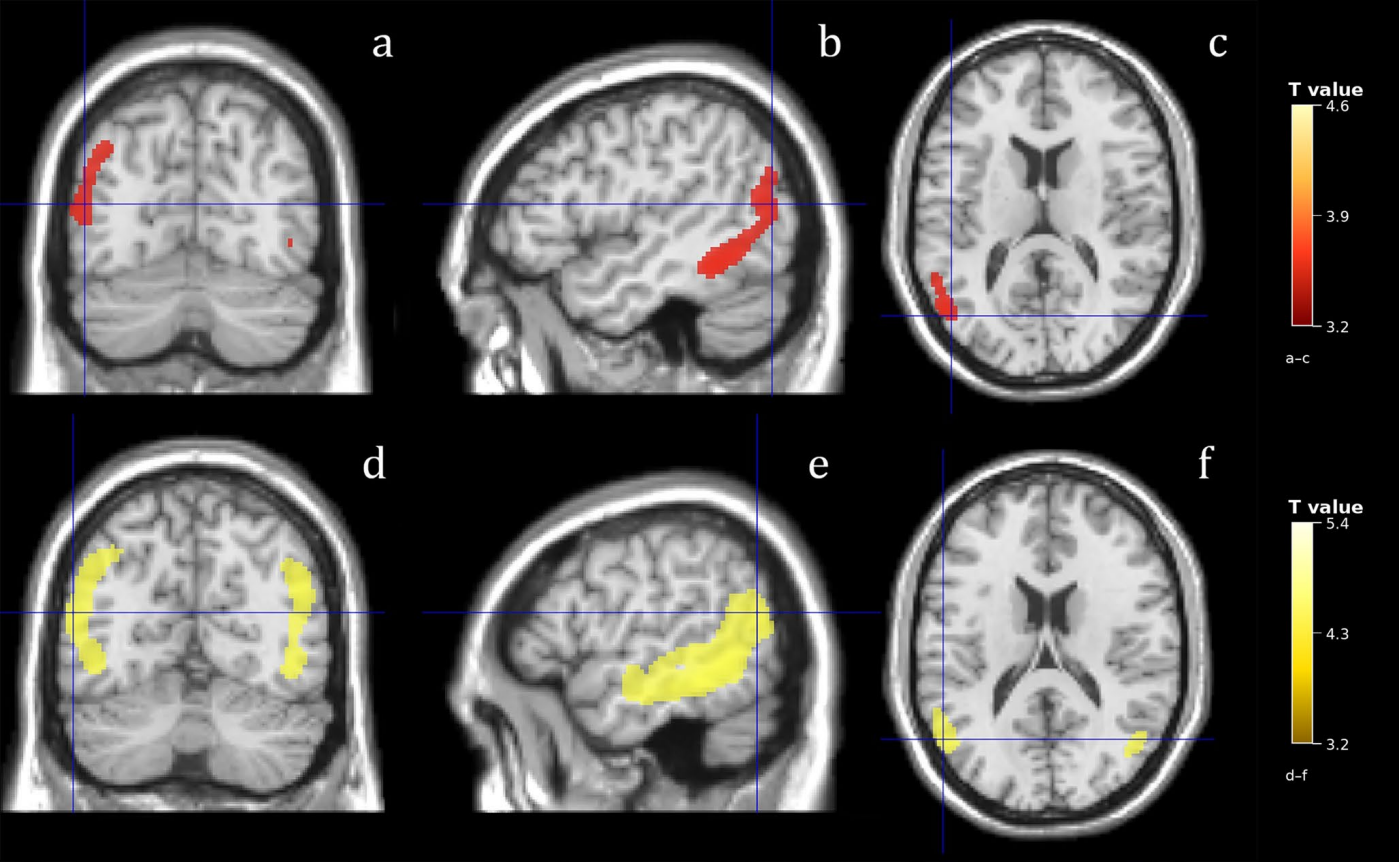
Voxel-wise cerebellum-normalised [^18F]FDG PET associations in the full study cohort. Statistical parametric maps show negative associations between plasma tau-related biomarkers and cerebellum-normalised regional [^18F]FDG uptake in the full cohort (*n* = 100), adjusted for age and sex. Panels a–c show plasma p-tau181, and panels d–f show plasma p-tau181/Aβ42. Results are displayed on a standard anatomical template using thresholded continuous T-value maps at voxel-level *p* < 0.001 uncorrected with a minimum cluster extent of k ≥ 100 voxels. The colour bars indicate T values

No suprathreshold clusters were detected for plasma p-tau217. Plasma p-tau217/Aβ42 showed one small negative cluster in the left ventral temporal region (kE = 207; peak T = 3.87; MNI coordinates − 54, −28, − 20). Full-cohort voxel-wise results are summarized in Table 2 and illustrated in Fig. 1.

**Table 2:** Voxel-wise associations between plasma biomarkers and cerebellum-normalised regional [^18F]FDG uptake in the full study cohort (*n* = 100). The table summarizes SPM25 multiple regression analyses performed on cerebellum-normalised [^18F]FDG SUVR images. For each model, the biomarker of interest was entered as the main regressor, with age and sex included as nuisance covariates. Results are displayed at voxel-level *p* < 0.001 uncorrected with a minimum cluster extent of k ≥ 100 voxels. Only suprathreshold clusters are reported. kE = cluster extent in voxels; MNI = Montreal Neurological Institute coordinates; SUVR = standardized uptake value ratio

Biomarker (contrast)	kE	Peak T	MNI coordinates (x, y, z)	Region
Plasma Aβ42/Aβ40 (+)	—	—	—	No suprathreshold clusters
Plasma Aβ42/Aβ40 (−)	—	—	—	No suprathreshold clusters
Plasma p-tau181 (+)	—	—	—	No suprathreshold clusters
Plasma p-tau181 (−)	1348	4.50	−48, − 46, −12	Left ventral temporal/fusiform cortex
Plasma p-tau181 (−)	433	4.49	44, − 54, −8	Right ventral temporal/fusiform cortex
Plasma p-tau181/Aβ42 (+)	—	—	—	No suprathreshold clusters
Plasma p-tau181/Aβ42 (−)	3340	5.31	−50, − 44, −14	Left ventral temporal/fusiform cortex
Plasma p-tau181/Aβ42 (−)	1883	4.93	52, − 44, −12	Right ventral temporal/fusiform cortex
Plasma p-tau217 (+)	—	—	—	No suprathreshold clusters
Plasma p-tau217 (−)	—	—	—	No suprathreshold clusters
Plasma p-tau217/Aβ42 (+)	—	—	—	No suprathreshold clusters
Plasma p-tau217/Aβ42 (−)	207	3.87	−54, − 28, −20	Left ventral temporal region

### Voxel-wise cerebellum-normalised analysis in the CSF-confirmed AD subgroup

The same voxel-wise cerebellum-normalised SPM approach was applied to the CSF-confirmed AD subgroup (*n* = 58), using age and sex as covariates. Given the smaller sample size, results were evaluated at voxel-level *p* < 0.01 uncorrected with a minimum cluster extent of k ≥ 1000 voxels.

For plasma Aβ42/Aβ40, one negative suprathreshold cluster was detected, with a peak in the right orbitofrontal/ventral frontal region (kE = 2083; peak T = 4.01; MNI coordinates 8, 30, − 22). This finding was not considered a primary tau-related result and was interpreted cautiously.

In the CSF-confirmed AD subgroup, the most consistent tau-related finding was observed for plasma p-tau181/Aβ42. Higher plasma p-tau181/Aβ42 was associated with lower cerebellum-normalised [^18F]FDG uptake in multiple suprathreshold clusters involving temporal/temporo-limbic and posterior cortical regions. The main clusters included peaks at 52, 4, − 28 (kE = 1156; peak T = 3.80), − 36, −4, − 36 (kE = 1477; peak T = 4.07), − 68, −68, 24 (kE = 3022; peak T = 3.38), 68, − 70, 4 (kE = 1013; peak T = 3.07), and 6, − 106, 32 (kE = 2569; peak T = 4.11).

No consistent cortical tau-related pattern was retained for plasma p-tau181 alone in the CSF-confirmed AD subgroup.

**Fig. 2:**
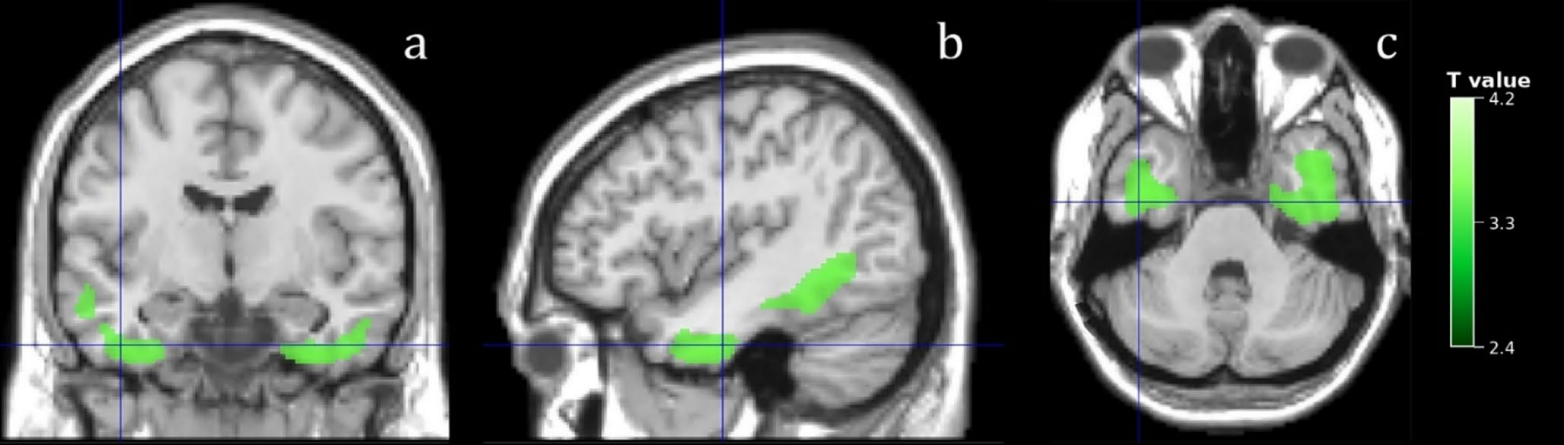
Voxel-wise cerebellum-normalised [^18F]FDG PET associations in the CSF-confirmed AD subgroup. Statistical parametric maps show negative associations between plasma p-tau181/Aβ42 and cerebellum-normalised regional [^18F]FDG uptake in the CSF-confirmed AD subgroup (*n* = 58), adjusted for age and sex. Results are displayed on a standard anatomical template using thresholded continuous T-value maps at voxel-level *p* < 0.01 uncorrected with a minimum cluster extent of k ≥ 1000 voxels. The colour bar indicates T values

No suprathreshold clusters were detected for plasma p-tau217 or plasma p-tau217/Aβ42 in the CSF-confirmed AD subgroup. Subgroup voxel-wise results are summarized in Table 3 and illustrated in Fig. 2.

**Table 3:** Voxel-wise associations between plasma biomarkers and cerebellum-normalised regional [^18F]FDG uptake in the CSF-confirmed AD subgroup (*n* = 58). The table summarizes SPM25 multiple regression analyses performed on cerebellum-normalised [^18F]FDG SUVR images. For each model, the biomarker of interest was entered as the main regressor, with age and sex included as nuisance covariates. Given the smaller sample size, results are displayed at voxel-level *p* < 0.01 uncorrected with a minimum cluster extent of k ≥ 1000 voxels. Only suprathreshold clusters retained for the main anatomical interpretation are reported. kE = cluster extent in voxels; MNI = Montreal Neurological Institute coordinates; SUVR = standardized uptake value ratio

Biomarker (contrast)	kE	Peak T	MNI coordinates (x, y, z)	Region
Plasma Aβ42/Aβ40 (+)	—	—	—	No suprathreshold clusters
Plasma Aβ42/Aβ40 (−)	2083	4.01	8, 30, − 22	Right orbitofrontal/ventral frontal region
Plasma p-tau181 (+)	—	—	—	No consistent cortical suprathreshold pattern retained for main anatomical interpretation
Plasma p-tau181 (−)	—	—	—	No consistent cortical suprathreshold pattern retained for main anatomical interpretation
Plasma p-tau181/Aβ42 (+)	—	—	—	No suprathreshold clusters
Plasma p-tau181/Aβ42 (−)	1156	3.80	52, 4, − 28	Right anterior temporal/temporo-limbic region
Plasma p-tau181/Aβ42 (−)	1477	4.07	−36, − 4, −36	Left inferior temporal/temporo-limbic region
Plasma p-tau181/Aβ42 (−)	3022	3.38	−68, − 68, 24	Left posterior temporo-parietal cortex
Plasma p-tau181/Aβ42 (−)	1013	3.07	68, − 70, 4	Right posterior temporal/occipito-temporal region
Plasma p-tau181/Aβ42 (−)	2569	4.11	6, − 106, 32	Posterior occipital cortex
Plasma p-tau217 (+)	—	—	—	No suprathreshold clusters
Plasma p-tau217 (−)	—	—	—	No suprathreshold clusters
Plasma p-tau217/Aβ42 (+)	—	—	—	No suprathreshold clusters
Plasma p-tau217/Aβ42 (−)	—	—	—	No suprathreshold clusters

## Discussion

The present voxel-wise analysis used cerebellum-normalised [^18F]FDG SUVR images to evaluate the relationship between plasma AD biomarkers and regional glucose metabolism, a functional marker commonly used to characterise AD-related neurodegeneration [20, 21]. This approach avoids whole-brain proportional scaling, which may be problematic in dementia [^18F]FDG PET studies because widespread cortical hypometabolism can influence the global mean and potentially alter regional statistical patterns. The most robust and spatially consistent finding was the negative association between plasma p-tau181/Aβ42 and regional [^18F]FDG uptake, predominantly involving bilateral ventral temporal/fusiform regions in the full study cohort and persisting, with a broader distribution, in the CSF-confirmed AD subgroup.

In the full cohort, higher plasma p-tau181/Aβ42 was associated with lower cerebellum-normalised [^18F]FDG uptake in bilateral ventral temporal/fusiform cortices. Plasma p-tau181 alone showed a similar but less extensive bilateral ventral temporal/fusiform pattern, whereas plasma p-tau217 was not associated with suprathreshold clusters and p-tau217/Aβ42 showed only a small focal cluster. These findings suggest that, in this cohort, amyloid-normalised p-tau181 was more closely related to regional metabolic dysfunction than p-tau181 alone or p-tau217-based measures. This is biologically plausible because the p-tau181/Aβ42 ratio integrates tau-related and amyloid-related information, potentially providing a stronger link with downstream neurodegenerative expression than either component alone [1, 6, 22].

The predominance of ventral temporal/fusiform involvement after cerebellar normalisation differs from the posterior medial emphasis commonly described in AD [^18F]FDG PET studies [20, 21]. This difference should not be interpreted as contradicting the established AD metabolic phenotype. Posterior cingulate and precuneus hypometabolism remain central features of AD, but the present cerebellum-normalised voxel-wise analysis identified the strongest plasma tau-related metabolic associations in ventral temporal/fusiform regions. These regions are vulnerable to AD-related neurodegeneration and may reflect a component of disease expression that becomes more apparent when regional uptake is referenced to cerebellar activity rather than to the whole-brain mean.

The CSF-confirmed AD subgroup provided a more biologically homogeneous framework but also had lower statistical power. In this subgroup, p-tau181/Aβ42 remained the most interpretable tau-related marker, showing negative associations with cerebellum-normalised [^18F]FDG uptake in temporal/temporo-limbic and posterior cortical clusters. Conversely, p-tau217 and p-tau217/Aβ42 showed no suprathreshold associations. These negative subgroup findings should not be interpreted as evidence that p-tau217 biomarkers are clinically uninformative. Plasma p-tau217 is strongly supported as a marker of AD pathology in diagnostic studies, including head-to-head comparisons with [^18F]FDG PET [7, 23]. However, its cross-sectional relationship with regional [^18F]FDG hypometabolism may be weaker or more difficult to detect within a smaller biomarker-confirmed AD subgroup, particularly when biological variance is restricted.

The finding that p-tau181/Aβ42, rather than p-tau217, showed the most consistent association with [^18F]FDG PET in this study also reinforces the distinction between biomarkers of AD pathology and imaging markers of neurodegenerative expression. Plasma phosphorylated tau markers are primarily intended to capture molecular AD pathology, whereas [^18F]FDG PET reflects regional synaptic and neuronal dysfunction. These two levels of information are related but not interchangeable. Prior work has similarly shown that plasma p-tau biomarkers can outperform [^18F]FDG PET for identifying biologically defined AD, whereas [^18F]FDG PET may remain more closely linked to clinical phenotype and functional impairment [6, 7]. Our results support this complementary interpretation.

The heterogeneous composition of the full study cohort deserves specific consideration. The cohort included patients with AD-related cognitive impairment as well as non-AD neurodegenerative and non-neurodegenerative conditions. Therefore, full-cohort plasma tau–[^18F]FDG associations should not be interpreted purely as within-AD biological gradients. They may partly reflect AD-versus-non-AD biological separation, because AD cases have higher plasma tau-related biomarker values and characteristic patterns of metabolic vulnerability. This is not necessarily a limitation of the full-cohort analysis, since the clinical setting in which plasma biomarkers and [^18F]FDG PET are used is inherently heterogeneous. However, it does affect interpretation: the full-cohort analysis is best viewed as reflecting biomarker–metabolism relationships across a real-world diagnostic spectrum, whereas the CSF-confirmed AD subgroup provides a more restricted assessment within biologically supported AD.

The plasma Aβ42/Aβ40 findings were not central in the present analysis. In the full cohort, plasma Aβ42/Aβ40 showed no suprathreshold voxel-wise associations after cerebellar normalisation. In the AD subgroup, a single negative orbitofrontal/ventral frontal cluster was observed; however, given the smaller subgroup size, the exploratory uncorrected statistical threshold, and the isolated nature of this finding, it should be interpreted cautiously and should not be considered a primary amyloid-related metabolic pattern. This is consistent with the notion that Aβ42/Aβ40 is more directly related to amyloid burden than to regional glucose metabolism. Previous studies have shown stronger links between plasma Aβ42/Aβ40 and amyloid measures than between plasma amyloid ratios and [^18F]FDG PET hypometabolism [24, 25].

Overall, the present analysis indicates that cerebellum-normalised voxel-wise [^18F]FDG PET associations with plasma biomarkers are selective. The most reproducible finding involved p-tau181/Aβ42, with negative associations in ventral temporal/fusiform regions in the full cohort and persisting associations in the CSF-confirmed AD subgroup. In contrast, p-tau217-based measures did not show robust cross-sectional voxel-wise [^18F]FDG PET associations after cerebellar normalisation. This does not diminish the diagnostic value of p-tau217 as a blood-based AD biomarker, but suggests that its relationship with regional metabolic impairment may require larger cohorts, longitudinal designs, or different disease-stage stratification to be fully characterised [26].

## Limitations

Several limitations should be acknowledged. First, this was a single-centre retrospective study with a modest sample size, particularly in the CSF-confirmed AD subgroup. Second, although cerebellar normalisation addresses an important limitation of whole-brain proportional scaling, the choice of reference region can itself influence [^18F]FDG PET results, and the cerebellum may not be entirely unaffected in all neurodegenerative conditions. Third, the full study cohort was diagnostically heterogeneous. This reflects real-world clinical practice but means that full-cohort associations may partly reflect AD-versus-non-AD separation rather than purely continuous within-disease relationships. Fourth, voxel-wise results were assessed using exploratory uncorrected thresholds; therefore, the findings should be interpreted as hypothesis-generating and require confirmation in larger independent cohorts. Fifth, the cross-sectional design prevents assessment of whether plasma tau-related biomarkers predict subsequent regional metabolic decline. Finally, although MMSE and education data were available descriptively, the present study was not designed to model detailed clinical progression or cognitive-domain-specific relationships with [^18F]FDG PET.

## Conclusions

Using voxel-wise cerebellum-normalised [^18F]FDG PET analysis, plasma p-tau181/Aβ42 showed the most robust association with regional hypometabolism, predominantly involving bilateral ventral temporal/fusiform regions in the full clinical cohort and persisting in the CSF-confirmed AD subgroup. Plasma p-tau181 showed a similar but less extensive full-cohort pattern, whereas p-tau217-based measures did not show robust cross-sectional voxel-wise associations after cerebellar normalisation. These findings support a complementary role of plasma tau biomarkers and [^18F]FDG PET, with plasma biomarkers indexing AD-related molecular pathology and [^18F]FDG PET providing information on regional metabolic dysfunction. Given the heterogeneity of the full cohort and the exploratory statistical thresholds, these results should be confirmed in larger independent and longitudinal cohorts.

## Supplementary information

Below is the link to the electronic supplementary material.

Supplementary File 1 (DOCX 477 KB)

## Data Availability

The datasets generated and/or analysed during the current study are available from the corresponding author on reasonable request.

## Code availability

Custom code/scripts used for image processing and statistical analyses are available from the corresponding author on reasonable request.
